# Performance of Artificial Intelligence Models in Radiographic Image Analysis for Predicting Hip and Knee Prosthesis Failure: A Systematic Review

**DOI:** 10.3390/bioengineering13010122

**Published:** 2026-01-21

**Authors:** Riccardo Stuani, Marco Di Maio, Vincenzo Di Matteo, Katia Chiappetta, Guido Grappiolo, Mattia Loppini

**Affiliations:** 1 Department of Biomedical Sciences, Humanitas University, Via Rita Levi Montalcini 4, 20090 Pieve Emanuele, Italydimaiomarco95@gmail.com (M.D.M.); vincenzo.dimatteo@humanitas.it (V.D.M.); 2 IRCCS Humanitas Research Hospital, Via Alessandro Manzoni 56, 20089 Rozzano, Italy; katia.chiappetta@humanitas.it (K.C.);

**Keywords:** AI, failure prediction, radiographic images, joint replacement

## Abstract

**Background and objectives**: The increasing volume of total hip and knee arthroplasty created a significant postoperative surveillance burden. While plain radiographs are standard, the detection of aseptic loosening is subjective. This review evaluates the state of the art regarding AI in radiographic analysis for identifying aseptic loosening and mechanical failure in primary hip and knee prostheses. **Methods**: A systematic search in PubMed, Scopus, Web of Science, and Cochrane was conducted up to November 2025, following PRISMA guidelines. Peer-reviewed studies describing AI tools applied to radiographs for detecting aseptic loosening or implant failure were included. Studies focusing on infection or acute complications were excluded. **Results**: Ten studies published between 2020 and 2025 met the inclusion criteria. In internal testing, AI models demonstrated high diagnostic capability, with accuracies ranging from 83.9% to 97.5% and AUC values between 0.86 and 0.99. A performance drop was observed during external validation. Emerging trends include the integration of clinical variables and the use of sequential imaging. **Conclusions**: AI models show robust potential to match or outperform standard radiographic interpretation for detecting failure. Clinical deployment is limited by variable performance on external datasets. Future research must prioritize robust multi-institutional validation, explainability, and integration of longitudinal data.

## 1. Introduction

Total hip and total knee arthroplasty (THA and TKA) represent highly successful and cost-effective surgical options for end-stage joint disease, with predictable gains in function and patient-reported quality of life [1]. As a result of their success and the aging global population, the volume of these procedures has risen exponentially [2]. Projections estimate that by 2030 and 2040, the annual volume of primary and revision arthroplasties will more than double in the United States and Europe [3,4]. This rise in surgical volume generates a parallel increase in the postoperative surveillance burden, threatening the sustainability of standard follow-up protocols and healthcare resources [5].

Despite high implant survivorship, a subset of patients requires revision surgery. While complications such as periprosthetic joint infection (PJI) and instability occur, aseptic loosening remains the leading cause of long-term failure and the primary target of routine radiographic surveillance [6]. Unlike acute complications, aseptic loosening is often insidious, developing slowly over years without immediate clinical symptoms, making its early detection heavily dependent on longitudinal imaging monitoring.

To manage this growing surveillance demand, “virtual clinic” models have been developed to triage patients effectively, reserving face-to-face consultations for those at high risk of failure [7,8]. The efficacy of these models relies heavily on the accurate interpretation of plain radiographs. However, manual interpretation has inherent limitations. The detection of early signs of aseptic failure, such as subtle radiolucent lines, osteolysis, or minor component migration, is subjective and prone to inter-observer variability [1]. Furthermore, the sensitivity of plain radiographs for detecting early loosening can be limited, potentially leading to delayed diagnoses and more complex revision surgeries [9]. With increasing workload pressures, orthopaedic surgeons may be at greater risk of overlooking subtle pathological features on 2D radiographs, motivating the development of automated, objective diagnostic support. In this context, artificial intelligence (AI), particularly deep learning (DL) based on convolutional neural networks (CNNs), has gained prominence in medical image analysis. Trained on large radiographic datasets, these methods can identify complex imaging signatures that may be challenging to appreciate on visual assessment, providing scalable support alongside clinician review [10]. Recent studies have demonstrated the potential of AI to automate implant identification, measure orientation parameters, and predict failure risk, thereby facilitating risk stratification and the implementation of virtual follow-up clinics [1,2].

While individual studies have explored the application of AI in arthroplasty, the literature regarding its specific diagnostic accuracy in this context remains fragmented. Most existing studies are limited to single-institution datasets, raising concerns about domain shifts, such as variations in X-ray acquisition protocols, scanner manufacturers, and patient demographics across different centres. Only recently have multicentre initiatives, such as the work by Wu et al. [11], begun to address these limitations by validating algorithms across diverse populations to ensure robustness. Furthermore, technical hurdles regarding integration into hospitals and the lack of prospective real-time validation continue to hinder the widespread adoption of these tools in daily orthopaedic practice. Therefore, the aim of this systematic review is to evaluate the current state of the art regarding the use of artificial intelligence in radiographic image analysis for specifically identifying and predicting aseptic loosening and mechanical failure in primary hip and knee prostheses.

## 2. Materials and Methods

### 2.1. Study Design

A systematic literature review was conducted with the aim of providing a summary of the use of AI-based tools applied to the radiographic detection of aseptic loosening and failure in hip and knee arthroplasty, identifying trends and limitations, and providing a framework to support future research. This study was carried out following the guidelines of the Preferred Reporting Items for Systematic Reviews and Meta-Analyses (PRISMA) [12].

### 2.2. Inclusion and Exclusion Criteria

Eligible studies were English-language, full-text, peer-reviewed articles describing the development or validation of AI-based tools applied to plain radiographs in humans for detecting aseptic loosening or arthroplasty failure following primary THA or TKA. No restrictions were placed on the year of publication.

Papers involving joints other than the hip or knee, partial arthroplasties, non-image-based approaches, studies that did not explicitly use AI, and studies that did not directly analyse radiographs were excluded. We also excluded work focused on non-aseptic failure causes (periprosthetic infection, fracture, dislocation) and non-primary research articles, including reviews, conference abstracts, and editorials. Studies focused on infection were excluded because their diagnosis heavily relies on clinical and laboratory biomarkers rather than solely on radiographic signs.

### 2.3. Literature Search Strategy

A comprehensive search was conducted in the PubMed, Scopus, Web of Science, and Cochrane Library databases to identify studies that used AI techniques in the development of tools applied to the radiographic diagnosis of prosthesis failure. The search strategy included the following keywords combined with Boolean operators: (“hip prosthesis” OR “hip arthroplasty” OR “knee prosthesis” OR “knee arthroplasty” OR “total hip replacement” OR “total knee replacement” OR “THA” OR “TKA” OR “hip implants” OR “knee implants”) AND (“artificial intelligence” OR “machine learning” OR “deep learning” OR “neural networks” OR “computer vision” OR “radiomics”) AND (“prosthesis failure” OR “implant failure” OR “failure” OR “prosthesis loosening” OR “implant loosening” OR “revision” OR “implant migration”).

The search was conducted on 26 November 2025, and was limited to titles and abstracts. Retrieved records were deduplicated prior to screening. Study selection was performed in two sequential stages by two reviewers working independently (M.D.M. and M.L.). First, titles and abstracts were screened to identify potentially eligible studies and exclude clearly irrelevant reports. Second, the full texts of the remaining articles were obtained and assessed against the predefined inclusion and exclusion criteria. Disagreements at any stage were resolved by discussion until a consensus was reached.

### 2.4. Synthesis of the Results

Data extracted from the selected studies were summarized to present their main methodological characteristics and the most relevant results. The aspects analysed included the study authorship and year, type of arthroplasty (hip or knee), dataset size and origin, AI model architecture employed, reference standard used for diagnosis, and performance metrics including accuracy, sensitivity, specificity, and Area Under the Curve (AUC). For the purpose of this review, “Aseptic Loosening” was defined as mechanical failure due to osteolysis or loss of fixation in the absence of infection. “General Failure” was defined as any complication requiring revision surgery (including instability, wear, or unspecified mechanical failure), excluding periprosthetic joint infection (PJI). Risk of bias was assessed using the Prediction model Risk Of Bias ASsessment Tool (PROBAST). The tool evaluates four domains: participants, predictors, outcome, and analysis. Studies were classified as low, high, or unclear risk based on study design, data handling, and the presence of external validation. A meta-analysis was not feasible since studies adopted a minimum set of reporting and methodological standards. Specifically, pooling would require a harmonized definition of the clinical task and failure endpoint, sufficient comparability of datasets (implant types, radiographic modality and protocol, sample size, inclusion and exclusion criteria, and class prevalence), and a transparent description of inputs and pre-processing (views acquired, segmentation approach, and any clinical covariates). In addition, studies should report performance at a predefined decision threshold. Finally, validation should follow comparable external testing procedures using independent cohorts that meet the same specifications.

## 3. Results

### 3.1. Study Selection and Characteristics

**Figure 1:**
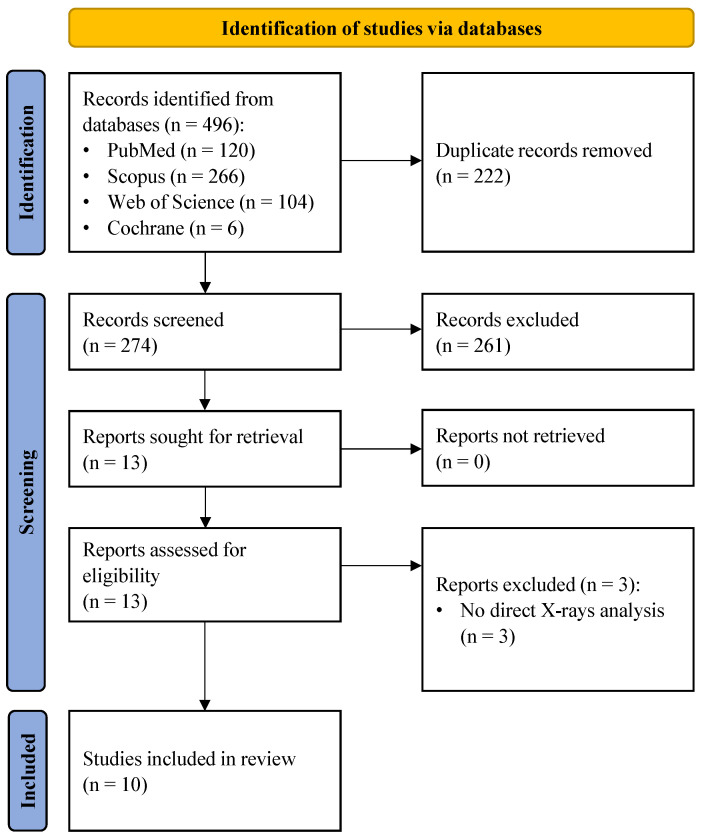
PRISMA (Preferred Reporting Items for Systematic Reviews and Meta-Analyses) flow diagram.

The systematic literature search initially yielded 496 records. Following the removal of duplicates and the screening of titles and abstracts, 13 full-text articles were assessed for eligibility. Three studies were excluded as they did not involve direct radiographic image analysis. Ultimately, 10 studies met the inclusion criteria and were included in the systematic review (Figure 1).

The characteristics of the included studies are summarized in Table 1. All articles were published between 2020 and 2025. Six studies focused exclusively on THA, while three focused on TKA. One study developed models for both joints. The datasets ranged in size from small cohorts of approximately 200 images to large multicentre datasets exceeding 2900 images. The majority of studies utilized CNNs, with DenseNet architectures being the most frequently employed model. The reference standard for establishing ground truth was consistently defined as intraoperative findings during revision surgery, expert consensus, or registry data. Features of the models used in each study, together with performance and AI-specific attributes, are reported in Table 2.

### 3.2. Prediction of Implant Failure

Among the included studies, a distinction can be made between those targeting specific aseptic loosening and those predicting all-cause failure. Six studies specifically developed algorithms to distinguish aseptic loosening from fixed implants or other specific aetiologies. These models generally demonstrated high diagnostic capability, often exceeding 90% accuracy.

Rahman et al. [20] developed “HipXNet”, a stacking ensemble model combining DenseNet201 with a Random Forest meta-learner. This approach achieved the highest reported performance for binary loosening detection in hips with an accuracy of 96.1% and sensitivity of 96.4%. Two recent studies addressed the challenge of differential diagnosis using multi-class models. Wu et al. [11] employed a dual-channel ensemble, analysing both anteroposterior (AP) and lateral views, trained on a large cohort. In the external validation cohort, this model achieved an accuracy of 82.6% and an AUC of 0.908 specifically for detecting aseptic loosening, effectively distinguishing it from infection and fracture. Similarly, Guo et al. [14] utilized a multi-branch ResNet18 network incorporating domain knowledge, reporting an accuracy of 88.1% and sensitivity of 89.7% for aseptic loosening in their internal validation.

For TKA, Lau et al. [16] trained an Xception-based model on 440 radiographs, reporting an accuracy of 96.3% and sensitivity of 96.1% for detecting loosening. Kim et al. [15] utilized a transfer learning approach with a VGG-19 architecture on a matched cohort of 200 patients (100 loose, 100 fixed), achieving an accuracy of 97.5% and sensitivity of 100% in identifying loosened knee implants.

Shah et al. [21] analysed a mixed cohort of hip and knee replacements (697 patients). Their image-only model achieved an accuracy of 70%, but the performance significantly improved to an accuracy of 88.3% and specificity of 95.6% when patient demographics and historical data were integrated into the model.

Four studies trained DL models to predict the broader outcome of implant failure, defined as the need for revision surgery. In these datasets, aseptic loosening was the predominant cause of failure, though the “failure” class also included cases of instability, wear, and unspecified mechanical failure.

Loppini et al. [17] achieved high performance using a DenseNet169 model on hip radiographs, reporting a test accuracy of 96.8% and an AUC of 0.993. Muscato et al. [19] combined DL features (DenseNet169) with a Support Vector Machine (SVM) classifier. While their internal validation showed an accuracy of 95.8%, the performance on a separate external cohort remained robust with an accuracy of 86.1% and an AUC of 0.961.

Corti et al. [13] applied a transfer learning strategy, adapting the hip failure model developed by Loppini et al. to predict knee arthroplasty failure. This cross-joint transfer learning approach yielded an internal test accuracy of 84.8%, which slightly decreased to 79.0% in the external validation cohort.

Masciulli et al. [18] introduced a novel approach using sequential radiographs to track implant evolution over time. Their model utilized a DenseNet121 combined with a Gated Recurrent Unit (GRU) to analyse a sequence of images (postoperative, intermediate, and pre-revision). This temporal analysis achieved high internal performance, although the accuracy dropped to 78.6% in the external validation.

**Figure 2:**
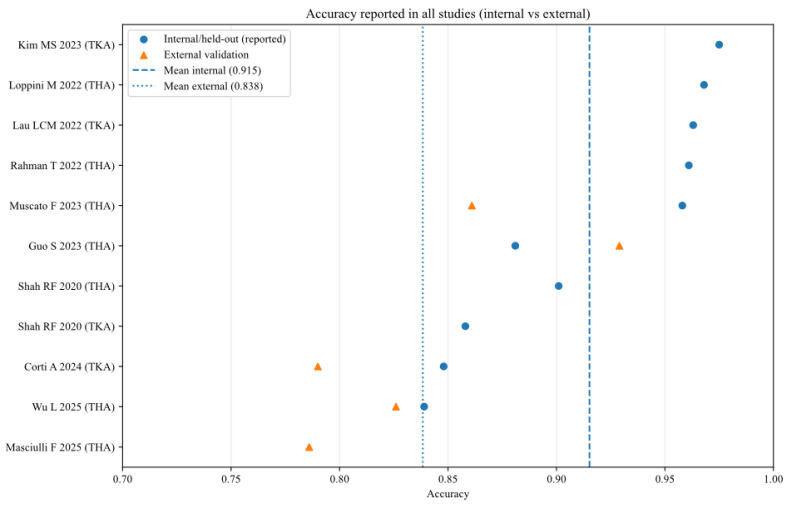
Accuracy of AI models for each study (internal and external validation datasets) [11,13,14,15,16,17,18,19,20,21].

**Figure 3:**
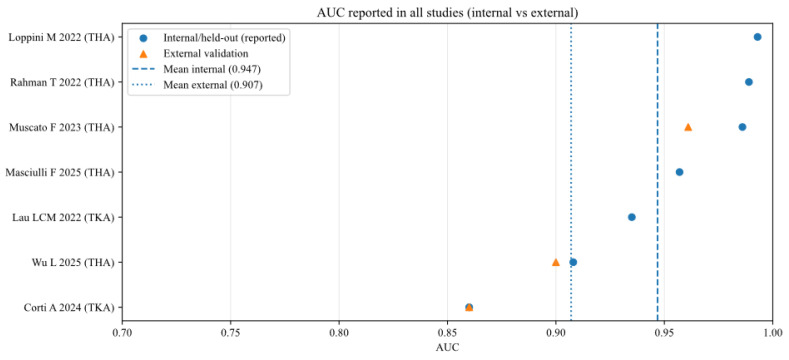
Area under the curve (AUC) of AI models for each study (internal and external validation datasets) [11,13,16,17,18,19,20].

**Figure 4:**
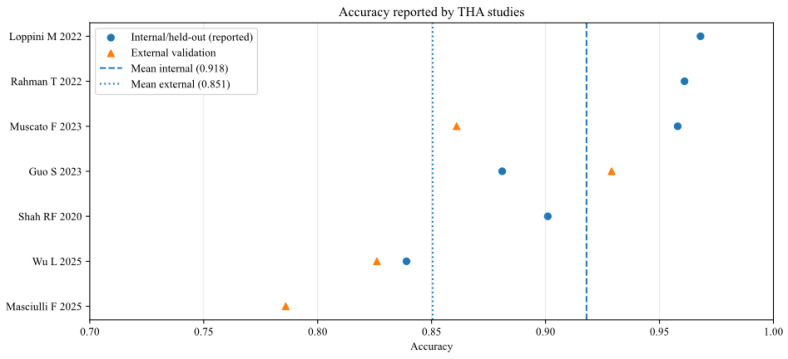
Accuracy of AI models for studies that analysed total hip arthroplasty (THA), reported in internal and external validation datasets [11,14,17,18,19,20,21].

**Figure 5:**
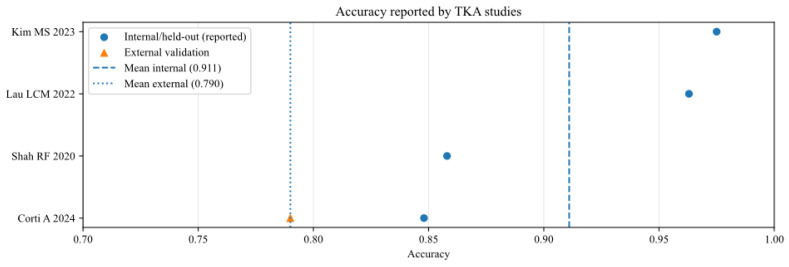
Accuracy of AI models for studies that analysed total knee arthroplasty (TKA), reported in internal and external validation datasets [13,15,16,21].

**Figure 6:**
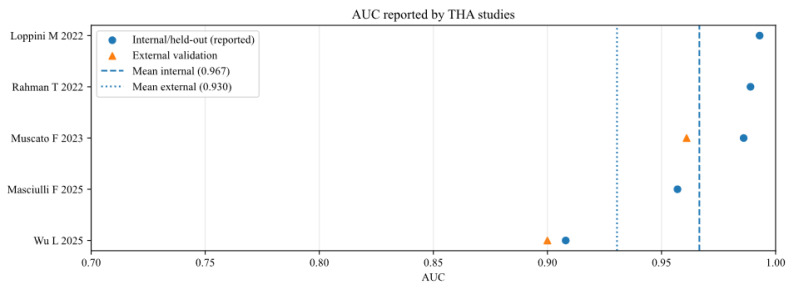
Area under the curve of AI models for studies that analysed total hip arthroplasty (THA), reported in internal and external validation datasets [11,17,18,19,20].

**Figure 7:**
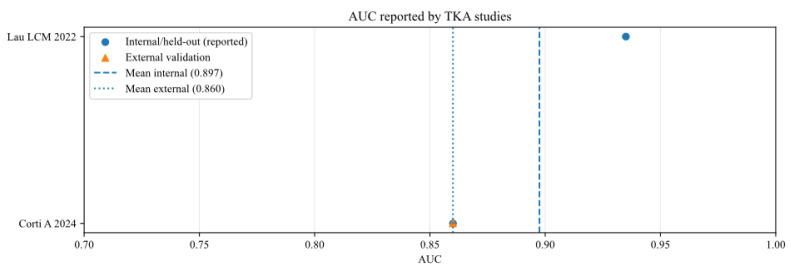
Area under the curve of AI models for studies that analysed total knee arthroplasty (TKA), reported in internal and external validation datasets [13,16].

To further analyse the robustness and generalizability of the included AI models, the reported performance metrics across studies were aggregated, stratifying by validation type (internal vs. external) and joint type (THA vs. TKA). Figure 2, Figure 3, Figure 4, Figure 5, Figure 6 and Figure 7 illustrate the distribution of accuracy and AUC for these subgroups. Consistent with the narrative synthesis, internal validation metrics generally exhibited higher and less variable values compared to external validation results. Furthermore, models developed for THA demonstrated slightly higher median performance metrics compared to those for TKA.

### 3.3. Risk of Bias Assessment

The risk of bias assessment according to PROBAST is detailed in Table 3. Only one study (Wu et al. [11]) was classified as having a low overall risk of bias. The primary source of bias across the included literature was the “Participants” domain (90% high or unclear risk). Most studies employed retrospective case–control designs with balanced datasets (e.g., 1:1 ratio of failed vs. non-failed implants), which do not reflect the true clinical prevalence of aseptic loosening and typically overestimate model performance. Regarding the “Analysis” domain, four studies were flagged as high risk due to the lack of external validation on a separate geographical cohort, relying solely on internal cross-validation or random splitting.

## 4. Discussion

The primary finding of this systematic review is that DL algorithms utilizing plain radiographs demonstrate high diagnostic performance for detecting aseptic loosening and implant failure in THA and TKA. Across the included retrospective studies, AI models achieved internal testing accuracies ranging from 83.9% to 97.5% and AUC values between 0.860 and 0.993. These results suggest that AI has the potential to match or even outperform current standard-of-care radiographic interpretation, offering a scalable solution to the growing surveillance burden in arthroplasty. From a clinical perspective, the sensitivity–specificity trade-off dictates the tool’s usage. High sensitivity is paramount for screening tools in virtual clinics to safely rule out loosening and avoid unnecessary visits, whereas high specificity is required for pre-surgical planning tools to rule in the need for revision and minimize false alarms.

Despite these promising metrics, a critical analysis of these results reveals a “generalizability gap”. Strong internal validation metrics often reflect dataset-specific characteristics rather than universal diagnostic capability. When models were tested on external cohorts, performance frequently dropped or became more variable. For instance, Corti et al. [13] observed a decrease in accuracy from 84.8% (internal) to 79.0% (external), and Masciulli et al. [18] reported an external accuracy of 78.6%, despite internal performance being consistently above 90%. This variability suggests that current models are sensitive to differences in scanners, imaging protocols, implant designs, and local prevalence rates, highlighting the need for robust multi-institutional validation before clinical deployment. Quantitatively, when comparing studies that reported both metrics, we observed a median performance drop in accuracy of approximately 9.5% (range: 5.8% to 17.2%) upon external validation. This phenomenon is largely attributable to domain shift, where the statistical distribution of training data differs from external cohorts due to scanner variability and protocol differences. To mitigate this, future studies should explore domain adaptation techniques or federated learning to train robust models across institutions without sharing patient data. Currently, only Wu et al. [11] have notably addressed this by employing a multicentre training approach rather than simple external testing.

The evidence base is currently characterized by significant heterogeneity in study design, preventing a quantitative meta-analysis. Two distinct methodological approaches were observed: models specifically trained to detect “aseptic loosening” and those predicting “general failure” or the need for revision. While general failure models yield high accuracy by including obvious pathologies like dislocation or fracture, specific loosening models are clinically more valuable for detecting subtle, early-stage osteolysis, where human error is most common. Furthermore, the definition of ground truth varied widely, ranging from robust intraoperative confirmation to less rigorous expert consensus or registry codes, which introduces variability in the reliability of the training labels.

The clinical utility of these models extends beyond simple accuracy. Future research must address the “black box” nature of deep learning to ensure trust. While some studies incorporated heatmaps (e.g., Grad-CAM), these visualizations can be misleading. Saliency maps often fail to capture semantic meaning, frequently acting as simple edge detectors that highlight high-contrast boundaries, such as the metal–bone interface, rather than the actual pathological feature. Consequently, there is a risk of confirmation bias, where clinicians may over-interpret vague heatmaps to fit a diagnosis. Therefore, rigorous validation is required to ensure they highlight plausible radiographic signs (e.g., radiolucent lines, osteolysis) rather than confounding artifacts. Wu et al. [11] provided a strong example of this by validating heatmaps against intraoperative findings. Additionally, reporting accuracy alone is insufficient for clinical translation. Future work should report calibration curves, decision thresholds, and clinical operating points (rule-out vs. rule-in) to demonstrate real-world value.

Architecturally, transfer learning with CNNs (DenseNet, VGG, and ResNet) remains the dominant approach. However, a significant shift towards longitudinal analysis is emerging. Aseptic loosening is inherently a progressive process, and single-timepoint analysis often fails to capture the subtle migration or osteolysis that precedes failure. Bulloni et al. [22] demonstrated the value of this temporal dimension by developing evolutionary radiological indices, quantifying changes in parameters like stem subsidence and radiolucency over the entire follow-up period. Their ML approach achieved an AUC of 0.94, reinforcing that analysing the rate of change is often more predictive than assessing a static change. Following this trend, other innovative methods such as ensemble learning and sequential models (CNN + GRU) are emerging to capture temporal progression, a key feature of loosening. By analysing the temporal evolution of an implant across multiple follow-up visits, Masciulli et al. [18] mimicked the clinical workflow of serial comparison, achieving high predictive values. This longitudinal approach aligns more closely with the standard orthopaedic workflow, where diagnosis is often confirmed by observing progressive changes over serial radiographs rather than a single snapshot. This suggests that future AI systems should move beyond single-snapshot analysis to incorporate longitudinal data, potentially enhancing sensitivity for progressive conditions like migration or subsidence. Additionally, the integration of clinical variables (e.g., age, BMI, time since surgery) was shown by Shah et al. [21] to significantly boost performance compared to image-only models, reinforcing that AI should function as a holistic decision-support tool rather than an isolated image reader.

### 4.1. Limitations

This review has several limitations that must be acknowledged. First, all included studies were retrospective in design, which introduces potential selection bias and may overestimate diagnostic accuracy compared to prospective clinical scenarios. Second, significant heterogeneity exists in outcome definitions (specific aseptic loosening vs. all-cause failure) and ground truth standards (revision surgery vs. expert consensus), preventing a quantitative statistical analysis. Third, several studies analysed data at the image level rather than the patient level, and they utilized small or class-imbalanced datasets (e.g., fewer loose implants than fixed ones). Without strict patient-level splitting, there is a risk of data leakage and optimistic performance estimates, and without rigorous stratification, the class imbalance can lead to overfitting, where the model favours the majority class, potentially inflating accuracy metrics while masking poor sensitivity. As highlighted by the PROBAST assessment, the majority of studies are at high risk of bias, primarily due to participant selection. The widespread use of balanced cohorts creates a spectrum bias, implying that the reported performance metrics are likely inflated compared to a real-world screening scenario where failure is a rare event. Finally, lack of prospective protocol registration may increase the risk of selective reporting.

### 4.2. Future Directions

To advance the field toward clinical implementation, future research must prioritize prospective, multicentre studies with harmonized definitions of ground truth and standardized imaging protocols. Subgroup analyses stratified by implant design (cemented vs. cementless) and time-from-index surgery are essential to determine if AI performs equally well across different clinical scenarios. Furthermore, studies should move beyond simple accuracy metrics to report decision curves and calibration plots, which are critical for determining clinical utility. Ultimately, head-to-head comparisons against expert readers in realistic clinical workflows will be essential to define the true value of AI as a decision-support tool. Finally, while the introduction highlights the potential of AI to support virtual follow-up clinics, future implementation studies are needed to systematically evaluate the clinical efficiency of such workflows in real-world practice.

## 5. Conclusions

AI has demonstrated robust capability in detecting aseptic loosening and hip or knee prosthesis failure from plain radiographs. However, the present evidence base is not yet sufficient for broad autonomous deployment due to generalizability gaps and ground truth variability. In terms of clinical decision-making, current results suggest a hybrid diagnostic strategy: AI models showing high sensitivity (e.g., DenseNet-based architectures) appear best suited as triage tools in “virtual clinics” to rule out loosening in asymptomatic patients, thereby prioritizing surgeon expertise for high-risk or symptomatic cases. Future efforts must focus on robust external validation, interpretability, and the integration of longitudinal data to develop reliable “glass-box” systems that can effectively support orthopaedic surgeons in managing the rising tide of arthroplasty surveillance.

## Figures and Tables

**Table 1:** Summary of general study characteristics. TKA: total knee arthroplasty; THA: total hip arthroplasty; BMI: body mass index; F: female.

Study	Country	Joint	Patients	Images (X-Ray)	Partitioning	Reference Standard	Demographics
**Corti A 2024 [13]**	Italy	TKA	Dev: 285 (150 failed, 135 non-failed) Ext: 969 (165 failed, 804 non-failed)	Dev: 602 (298 failed, 304 non-failed) Ext: 1937 (329 failed, 1608 non-failed)	80% (70:30 Train & Val) 20% Internal Test External Validation	Revision surgery	NR
**Guo S 2023 [14]**	China	THA	NR	443 failed (355 Training, 88 Testing)	5-Fold Cross Validation	Expert consensus	NR
**Kim MS 2023 [15]**	South Korea	TKA	200 (100 loosened, 100 fixed)	200 (100 loosened, 100 fixed)	80% Training 20% Test	Intraoperative findings	Loose: 70.4 yrs, 80% F, BMI 26.3 Fixed: 70.9 yrs, 80% F, BMI 26.5
**Lau LCM 2022 [16]**	Hong Kong	TKA	440 (206 loosened, 234 fixed)	440 (206 loosened, 234 fixed)	75% Test 25% Validation	Intraoperative findings	NR
**Loppini M 2022 [17]**	Italy	THA	630 (420 failed, 210 non-failed)	1853 (922 failed, 931 non-failed)	63% Training, 27% Validation 10% Test	Revision surgery	Age: 72 yrs Female 60%
**Masciulli F 2025 [18]**	Italy	THA	Dev: 205 (76 failed, 129 non-failed) Ext: 14 (7 failed, 7 non-failed)	Dev: 2291 Ext: 42	75% Training 25% Test External Validation	Revision surgery	Dev failed: 62 yrs, 34.4% F Dev non-failed: 66 yrs, 43.8% F
**Muscato F 2023 [19]**	Italy	THA	Dev: 280 (140 failed, 140 non-failed) Ext: 352 (275 failed, 77 non-failed)	Dev: 560 Ext: 771 (589 failed, 182 non-failed)	80% Training, 20% Validation External Validation	Revision surgery	Dev: 66 yrs, 63% F Ext: 67 yrs, 64% F
**Rahman T 2022 [20]**	Qatar	THA	NR	200 (112 loose, 94 fixed)	5-Fold Cross Validation 70% Training, 10% Validation 20% Test	Research results	NR
**Shah RF 2020 [21]**	USA	THA TKA	697 (222 loosened, 475 fixed) THA: 343 (85 loose, 258 fixed) TKA: 354 (137 loose, 217 fixed)	NR	60% Training, 20% Validation 20% Test	Intraoperative findings	Loose: 69.2 yrs, 55.9% F Fixed: 67.2 yrs, 53.9% F
**Wu L 2025 [11]**	China	THA	Dev: 1024 Ext: 402	2908	80% Training & Validation 20% Internal Test External Validation	Intraoperative findings	Train: 60 yrs, 54% F Test: 59 yrs, 53.2% F Ext: 66 yrs, 45.8% F

**Table 2:** Summary of AI models used in each study and best performances. AP: anteroposterior; TKA: total knee arthroplasty: GRU: gated recurrent unit; SVM: support vector machine; THA: total hip arthroplasty; AUC: area under the curve; SHAP: Shapley additive explanations.

Study	Input Data	Best AI Model	Specific Outcome	Best Performance	Explainability
**Corti A 2024 [13]**	AP + Lateral X-rays (Post primary TKA)	DenseNet169 (Transfer Learning from Hip model)	General failure	Val: Acc 89.9, Sens 93.0, Spec 87.0, AUC 93.8 Test: Acc 84.8, Sens 80.0, Spec 89.5, AUC 86.0 Ext: Acc 79.0, Sens 80.0, Spec 78.0, AUC 86.0	No
**Guo S 2023 [14]**	Single X-ray	Multi-branch ResNet18	Aseptic Loosening	Int: Acc 88.1, Sens 89.7, Precision 92.9, F1 91.2 Ext: Acc 92.9, Sens 93.1, Precision 96.4, F1 94.7	No
**Kim MS 2023 [15]**	Single AP X-ray	VGG-19	Aseptic Loosening	Int: Acc 97.5, Sens 100, Spec 95	No
**Lau LCM 2022 [16]**	Single X-rays	Xception	Aseptic Loosening	Int: Acc 96.3, Sens 96.1, Spec 90.9, AUC 93.5	Grad-CAM
**Loppini M 2022 [17]**	AP + Lateral X-ray	DenseNet169	General failure	Int Test: Acc 96.8, Sens 96.8, Spec 96.8, AUC 99.3	Grad-CAM
**Masciulli F 2025 [18]**	Sequential AP X-rays (2 or 3 per patient)	DenseNet + GRU	General failure	Int: Sens 91.7, Spec 90.3, F1 93.3, AUC 95.7 Ext: Acc 78.6, Sens 78.6	No
**Muscato F 2023 [19]**	AP + Lateral X-ray 3-image model (original, acetabulum, stem)	Hybrid ensemble (DenseNet169 + SVM classifier)	General failure	Int: Acc 95.8, Sens 96.8, Spec 94.8, F1 95.8, AUC 98.6 Ext: Acc 86.1, Sens 91.9, Spec 86.3, F1 87.4, AUC 96.1	SHAP analysis
**Rahman T 2022 [20]**	Single AP X-ray	DenseNet201 Stacking approach using Random forest	Aseptic Loosening	DenseNet201: Acc 94.7, Sens 94.7, Spec 94.5, F1 94.7, AUC 97.7 RF: Acc 96.1, Sens 96.4, Spec 96.7, F1 96.4, AUC 98.9	Score-CAM
**Shah RF 2020 [21]**	AP + Lateral X-ray Demographic & comorbidity	DenseNet	Aseptic Loosening	Overall: Acc 88.3, Sens 70.2, Spec 95.6 TKA only: Acc 85.8, Sens 69.8, Spec 95.2 THA only: Acc 90.1, Sens 70.3, Spec 94.6	No
**Wu L 2025 [11]**	AP + Lateral X-ray	Hip-Net: dual-channel ensemble (4 CNNs: VGG16, Inception-v3, ResNet-50, DenseNet-121)	Multiclass Classification (Aseptic Loosening)	Aseptic Loosening Int: Acc 83.9, Sens 83.2, Spec 84.4, AUC 90.8 Aseptic Loosening Ext: Acc 82.6, Sens 84.6, Spec 81.0, AUC 90.0	Grad-CAM

**Table 3:** Risk of bias according to PROBAST Domain.

Study	Participants	Predictors	Outcomes	Analysis	Overall
**Corti A 2024** [13]	High	Low	Low	Low	High
**Guo S 2023** [14]	High	Low	High	High	High
**Kim MS 2023** [15]	High	Low	Low	High	High
**Lau LCM 2022** [16]	High	Low	Low	High	High
**Loppini M 2022** [17]	High	Low	Low	Low	High
**Masciulli F 2025** [18]	High	Low	Low	Low	High
**Muscato F 2023** [19]	High	Low	Low	Low	High
**Rahman T 2022** [20]	High	Low	Unclear	High	High
**Shah RF 2020** [21]	Unclear	Low	Low	High	High
**Wu L 2025** [11]	Low	Low	Low	Low	Low

## Data Availability

The original contributions presented in the study are included in the article, further inquiries can be directed to the corresponding author.
